# The Association Between Exposure to Acrylamide and Mortalities of Cardiovascular Disease and All-Cause Among People With Hyperglycemia

**DOI:** 10.3389/fcvm.2022.930135

**Published:** 2022-07-18

**Authors:** Huanyu Wu, Xinyi Sun, Hongyan Jiang, Cong Hu, Jiaxu Xu, Changhao Sun, Wei Wei, Tianshu Han, Wenbo Jiang

**Affiliations:** ^1^National Key Discipline, Department of Nutrition and Food Hygiene, School of Public Health, Harbin Medical University, Harbin, China; ^2^Department of Pharmacology, College of Pharmacy, Harbin Medical University, Harbin, China; ^3^Department of Cardiology, The First Affiliated Hospital of Harbin Medical University, Harbin, China

**Keywords:** CVD mortality, all-cause mortality, hyperglycemia, NHANES, acrylamide

## Abstract

**Background:**

Acrylamide is a common environmental volatile organic compound that humans are frequently exposed to in their daily lives. However, whether exposure to acrylamide is associated with long-term survival in patients with hyperglycemia remains largely unknown.

**Methods and Results:**

A total of 3,601 hyperglycemic people were recruited in this study, including 1,247 people with diabetes and 2,354 people with pre-diabetes, who enrolled in the National Health and Nutrition Examination survey (2003–2004, 2005–2006, and 2013–2014). The acrylamide exposure was measured by the serum hemoglobin adduct of acrylamide (HbAA) and glycidamide (HbGA), and the ratio of HbAA and HbGA (HbAA/HbGA) was calculated, which were all categorized into quintiles. The National Death Index was used to identify the participants' death information until 2015. Cox proportional hazards (CPHs) regression models were performed to examine the survival relationship between these biomarkers and mortality. During the 28,652 person-year follow-up, 268 deaths due to the cardiovascular disease (CVD) were documented. After adjustment for multiple confounders, compared with participants in the lowest quintile of HbAA/HbGA, the participants in the highest quintile were more likely to die due to CVD (hazard ratio [*HR*] = 1.61, 95% *CI*: 1.09–2.39) and all-cause (*HR* = 1.59, 95% *CI*: 1.25–2.01). Moreover, subgroup analysis showed that the highest quintile of HbAA/HbGA in the people with diabetes or pre-diabetes was related to mortalities risk of CVD (*HR*_diabetes_ = 1.92, 95% *CI*: 1.11–3.31; *HR*_pre−diabetes_ = 1.78, 95% *CI*: 1.01–3.14) and all-cause mortality (*HR*_diabetes_ = 1.81, 95% *CI*: 1.27–2.58; *HR*_pre−diabetes_ = 1.59, 95% *CI*: 1.14–2.20). Additionally, no significant association between the levels of HbAA or HbGA and CVD mortality was observed among people with diabetes or pre-diabetes.

**Conclusion:**

Higher levels of HbAA/HbGA are associated with greater mortalities of CVD and all-cause among hyperglycemic people.

## Introduction

Acrylamide is an environmentally volatile organic compound, and human beings are frequently exposed to it in their everyday life (1). Acrylamide can be found in many aspects of daily life, such as daily cosmetics, textiles and plastic, cigarettes, water purification, and dietary intakes including various heated foods, beverages, potato crisps, and coffee (2–4). In addition to the neurotoxicity and carcinogenic toxicity of high concentrations of acrylamide, a few *in vivo* and *in vitro* studies have found that long-term exposure to low concentrations of acrylamide could induce hepatic oxidative stress and disturb lipid metabolism in rats (5, 6), and could also stimulate the inflammatory response and cell proliferation in HepG2 cells (7). Moreover, recent population-based studies found that serum biomarkers of acrylamide exposure were associated with hyperglycemia and dyslipidemia, prompting the conclusion that daily exposure to acrylamide probably influences the daily glucose control among people with hyperglycemia (8, 9). In addition, an association between acrylamide exposure and a higher risk of cardiovascular disease (CVD) was demonstrated in a population-based study (10). However, it is still largely unknown whether exposure to acrylamide is associated with long-term survival among people with hyperglycemia.

Evidence from animal studies has demonstrated that rats exposed to acrylamide could disrupt glucose homeostasis, induce β-cell dysfunction, and reduce the area of the aortic vessel and acetylcholine to endothelial-dependent arterial diastolic reactions (11, 12). Moreover, *in vitro* experiments revealed that chronic exposure to acrylamide could induce accelerated endothelial aging, even at low concentrations (13). These mechanisms are highly overlapping with the effect of hyperglycemia on the development of CVD. Based on this evidence, we, therefore, hypothesize that exposure to acrylamide is associated with an increased risk of CVD mortality among people with hyperglycemia. To examine this hypothesis, we examined the association of serum biomarkers of acrylamide with mortalities of CVD and all-cause in the participants with hyperglycemia using the national representative sample from the National Health and Nutrition Examination Survey (NHANES).

## Materials and Methods

### Study Population

The NHANES is a stratified, multilevel study using a national population sample in the United States, and its detailed information has been provided elsewhere (14). The present study included 3,601 hyperglycemic participants, consisting of 1,247 diabetes and 2,354 pre-diabetes, who were over 18 years old and measured the serum biomarkers of acrylamide. The participants who had missing information on serum acrylamide metabolites, mortality of all-cause, and CVD, and questionnaire were excluded. The NHANES program was approved by the National Health Statistics Research Ethics Committee, and all informed consent was provided by participants.

### Definition of Diabetes and Pre-diabetes

According to the American Diabetes Association, diabetes was defined as conforming to at least one of the following criteria: fasting blood glucose (FBG) > 7.0 mmol/L; 2-h postprandial blood glucose > 11.1 mmol/L; HbA1c > 6.5%; or a random plasma glucose > 11.1 mmol/L. Pre-diabetes was determined as meeting at least one of the criteria created by: FBG > 5.6 and <7.0 mmol/L; 2-h postprandial blood glucose > 7.8 and <11.1 mmol/L; or HbA1c > 5.7% and <6.5%.

### Measurements of Serum Biomarkers of Acrylamide

The N-terminal hemoglobin adducts of acrylamide (HbAA) and hemoglobin glycidamide (HbGA) in 300 μl of human whole blood or erythrocytes were quantified using high-performance liquid chromatography/tandem mass spectrometry (HPLC/MS/MS) based on a modified Edman reaction (15). The well-established procedure was briefly illustrated as follows: using pentafluorophenyl isothiocyanate as an Edman reagent, two adducts were isolated from the protein chain by modified Edman reaction (16). After the modified EDMAN degradation reaction, liquid–liquid extraction was performed, and the results were analyzed based on HPLC/MS/MS. The calibrator, reagent blank, and quality control materials were pretreated in the same way as the samples. Each sample was performed with two independent repeated measurements. The limits of the detection of HBbaA and HbGA were 3 and 4 pmol/g, respectively.

### Main Exposure and Main Outcome

The main exposures in our study were HbAA and HbGA levels in whole blood and the ratio of HbAA and HbGA (HbAA/HbGA). In addition, the outcome was the status of mortality according to the National Death Index (NDI) until the year 2015. The NDI is a reliable death identification resource. The disease-specific death was defined as International Classification of Diseases (ICD)-10 and the death due to CVD was determined as ICD-10 codes (I00-I09, I11, I13, I20-I51, and I60-I69). In summary, a total of 268 deaths (132 in diabetes and 136 in pre-diabetes) due to CVD and 709 deaths (332 in diabetes and 377 in pre-diabetes) due to all-cause were documented during the 28,652 person-year follow-up.

### Covariates Assessment

The covariates in our study included age (years), sex (women/men), race/ethnicity (Mexican American/non-Hispanic white/non-Hispanic black/other), smoking (yes or no), drinking status (yes or no), education levels (less than high school education and high school and above), annual household income (more than $100,000/$45,000–$75,000/$20, 000-$45,000/ < $20,000), occupation, body mass index (BMI, kg/m^2^), energy (kcal), exercise (yes or no), mean blood pressure (mmHg), whether to use insulin hypoglycemic tablets (yes or no), whether to take medication for hypertension (yes or no), and whether to take medication for cholesterol (yes or no).

### Statistical Analyses

Demographic and anthropometric characteristics were presented using the mean and standard deviation (SD) for the continuous variables and the number and percentage for categorical variables. The general linear model adjusted for age and sex was used to compare the differences in the biomarkers of acrylamide by different baseline characteristics. All statistical analyses were conducted by R 4.0.2 software, and all tests were two-sided. The value of *p* < 0.05 was considered statistically significant.

### Cox Proportional Hazards (CPH) Models

**Cox proportional hazards** (CPHs) models were conducted to calculate hazard ratios (*HR*s) and 95% *CI* for all-cause and CVD mortalities. The time scale in the Cox model used for the follow-up time was obtained by person-months from the date of the interview to their death, or the end of 2015, which was used for the time scale this Cox model. The HbAA, HbGA, and HbAA/HbGA were respectively categorized into quintiles, and the lowest quintile is regarded as the reference group. The confounders in the CPH model included age, sex, race, smoking status, drinking status, exercise, energy, education level, occupation, annual household income, BMI, mean blood pressure, whether to take medication for diabetes, whether to take medication for hypertension, and whether to take medication for cholesterol.

## Results

### Participant Characteristics

The differences in the whole blood HbAA, HbGA, and HbAA/HbGA levels according to the baseline characteristics are presented in Table 1. The results of the normality test showed that HbAA, HbGA, and HbAA/HbGA levels were non-normally distributed (Supplementary Figure 1). The participants with higher HbAA levels were younger, men, non-Hispanic blacks, with a lower use rate of medication for glucose and blood pressure, and higher use of medication for cholesterol, and higher smoking rate, drinking, and energy intake (all the *p* < 0.05). Similarly, the participants with higher HbGA levels were younger, Mexican American, with a lower use rate of medication for blood pressure and higher use of medication for cholesterol, and higher rates of smoking, drinking, and energy intake (all the *p* < 0.05). Additionally, the participants with obesity, diabetes, or hypertension had lower levels of HbAA, and the participants with hypertension had lower levels of HbGA, respectively (all the *p* < 0.05). Meanwhile, the participants with higher HbAA/HbGA levels were men, non-Hispanic blacks, with lower medication use for glucose and cholesterol, higher medication use for blood pressure, and higher rates of smoking, drinking, and energy intake. The ratio of HbAA/HbGA was significantly lower in obese, diabetes, and dyslipidemia participants (*p* < 0.05).

**Table 1:** The hemoglobin biomarkers of hemoglobin adduct of acrylamide (HbAA), hemoglobin adduct of glycidamide (HbGA), and HbAA/HbGA are grouped by various characteristics of the total hyperglycemia population.

**Characteristics**	**HbAA (pmol/g Hb)**		***P*_**fortrend**_**	**HbGA (pmol/g Hb)**		***P*_**fortrend**_**	**HbAA/HbGA**		***P*_**fortrend**_**
	**Medians**	**IQRs**		**Medians**	**IQRs**		**Medians**	**IQRs**	
Age			<0.001			<0.001			0.4
<60 years	57.4	(42.0, 102.0)		54.1	(37.8, 81.5)		1.15	(0.93, 1.45)	
≥ 60 years	47.1	(36.4, 64.6)		43.0	(31.5, 60.4)		1.15	(0.94, 1.41)	
Sex			<0.001			0.13			<0.001
Male (%)	53.9	(39.4, 88.7)		47.6	(33.2, 70.5)		1.22	(1.00, 1.49)	
Female (%)	49.5	(37.6, 72.8)		49.0	(35.3, 70.2)		1.07	(0.88, 1.31)	
Race			<0.001			<0.001			<0.001
Mexican American	53.4	(42.0, 70.7)		52.8	(39.7, 70.6)		1.04	(0.88, 1.27)	
Non-hispanic White	51.5	(38.7, 81.3)		48.9	(34.5, 72.9)		1.14	(0.92, 1.39)	
Non-hispanic Black	53.5	(37.2, 100.5)		44.2	(30.6, 66.0)		1.31	(1.06, 1.67)	
Others	45.4	(33.1, 69.2)		42.2	(33.8, 67.4)		1.10	(0.92, 1.29)	
Current smoking			<0.001			<0.001			<0.001
Never	45.0	(35.5, 57.4)		42.0	(31.5, 56.2)		1.09	(0.89, 1.30)	
Active	114.0	(74.3, 164.0)		79.9	(55.5, 115.0)		1.36	(1.12, 1.69)	
Current drinking			<0.001			<0.001			<0.001
Never	46.9	(36.6, 65.4)		46.0	(33.1, 63.5)		1.08	(0.88, 1.31)	
Current	54.5	(39.6, 89.6)		48.8	(34.3, 73.2)		1.19	(0.97, 1.47)	
College graduate or above			0.2			0.11			0.3
< High school	53.7	(39.2, 83.4)		49.7	(35.1, 72.1)		1.15	(0.93, 1.43)	
≥High school	51.0	(38.2, 77.3)		47.6	(33.6, 69.0)		1.15	(0.94, 1.42)	
Income			0.12			0.043			0.6
<10,000	52.1	(38.4, 85.1)		48.8	(34.2, 73.3)		1.16	(0.93, 1.43)	
≥10,000	51.1	(38.5, 73.1)		47.7	(33.7, 66.9)		1.15	(0.94, 1.41)	
Energy			<0.001			<0.001			<0.001
<1,829 kcal	49.7	(37.5, 72.3)		46.6	(33.2, 67.0)		1.14	(0.92, 1.38)	
≥1,829 kcal	53.9	(39.7, 90.7)		49.5	(35.1, 73.6)		1.17	(0.95, 1.46)	
Regular exercise			0.7			0.027			<0.001
Yes	51.3	(39.7, 70.9)		49.5	(36.4, 69.0)		1.10	(0.90, 1.34)	
No	52.0	(37.8, 85.7)		47.6	(33.2, 70.3)		1.18	(0.96, 1.46)	
Obesity			<0.001			0.6			<0.001
Yes	49.8	(37.7, 73.4)		47.9	(37.7, 95.6)		1.10	(0.91, 1.34)	
No	54.8	(39.8, 95.6)		49.2	(39.8, 95.6)		1.23	(1.00, 1.53)	
Diabetes			<0.001			0.060			<0.001
Yes	49.7	(37.0, 72.0)		47.2	(34.4, 68.4)		1.11	(0.89, 1.35)	
No	52.9	(39.5, 85.1)		48.4	(33.4, 73.6)		1.18	(0.96, 1.46)	
Hypertension			<0.001			<0.001			0.2
Yes	48.6	(36.7,71.7)		45.1	(32.4, 64.5)		1.15	(0.93, 1.40)	
No	55.3	(40.8,88.4)		51.6	(36.1, 75.2)		1.16	(0.94, 1.44)	
Dyslipidemia			0.5			<0.001			<0.001
Yes	52.1	(38.1, 87.1)		51.1	(35.3, 76.8)		1.11	(0.89, 1.36)	
No	51.5	(38.5, 77.3)		47.2	(33.5, 68.4)		1.17	(0.95, 1.44)	
Take medication for diabetes			<0.001			0.2			<0.001
Yes	49.3	(36.7, 69.8)		47.6	(33.0, 67.0)		1.10	(0.88, 1.33)	
No	52.4	(39.1, 84.0)		48.3	(34.3, 71.2)		1.17	(0.95, 1.45)	
Take medication for hypertension			<0.001			<0.001			0.027
Yes	47.5	(36.4, 67.7)		44.4	(32.3, 62.3)		1.15	(0.92, 1.38)	
No	55.1	(38.9, 102.0)		48.5	(32.0, 85.5)		1.14	(0.98, 1.46)	
Take medication for cholesterol			<0.001			0.007			0.001
Yes	49.5	(37.8, 71.3)		46.6	(33.4, 67.0)		1.13	(0.91, 1.38)	
No	46.1	(35.6, 61.5)		43.9	(31.0, 60.8)		1.16	(0.88, 1.39)	

*Continuous variables are presented as medians (IQRs). Categorical variables are presented as percentages. Diabetes is defined by FBG ≥ 7.0 mmol/L; 2-h PBG ≥ 11.1 mmol/L, random plasma glucose ≥ 11.1 mmol/L, or HbA1c ≥ 6.5%. Pre-diabetes is determined as meeting at least one of the criteria created by: 2-h PBG ≥ 7.8 and <11.1 mmol/L; FBG ≥ 5.6 and <6.9 mmol/L, or intermediate HbA1c ≥ 5.7% and <6.4%*.

### Acrylamide Exposure and Mortality

The relationships of HbAA, HbGA, and HbAA/HbGA in whole blood with CVD and all-cause mortalities among people with hyperglycemia are presented in Table 2. Compared with those in the lowest quintile of HbAA/HbGA, the participants in the highest quintile were more prone to die due to CVD and all-cause, with the *HR*s (95% *CI*) being 1.61 (1.09–2.39) and 1.59 (1.25–2.01), respectively, and sex was not a significant effect modifier of the above association (*p*_effectmodificationwithsex_ > 0.05). Meanwhile, compared with the participants in the lowest quintile of HbAA, the participants in the highest quintile of HbAA had a greater risk of CVD mortality (*HR*: 1.84, 95% *CI*: 1.00–3.37) and all-cause mortality (*HR*: 2.21, 95% *CI*: 1.46–3.05). Moreover, no significant association between quintiles of HbGA with CVD mortality was observed.

**Table 2:** Adjusted hazard ratios (*HR*s) for the associations between HbAA, HbGA, HbAA/HbGA and CVD or all-cause mortality in the total hyperglycemia population.

	**CVD mortality**		**All-cause mortality**	
	**Case/*N***	**HR (95%CI)**	**Case/*N***	**HR (95%CI)**
**HbAA(pmoL/g Hb)**			
Q1(<36.2)	79/722	1	182/722	1
Q2(36.2–46.3)	54/723	0.74(0.51–1.07)	159/723	0.95(0.76–1.19)
Q3(46.3–59.0)	47/719	0.73(0.49–1.11)	130/719	0.88(0.68–1.13)
Q4(59.0–95.7)	51/718	1.16(0.73–1.85)	127/718	1.18(0.88–1.59)
Q5(>95.7)	37/719	1.84(1.00–3.37)	111/719	2.21(1.46–3.05)
*P* for trend		0.002		<0.001
*P*_sex*HbAA_		0.018		0.222
**HbGA(pmoL/g Hb)**			
Q1(<31.5)	67/726	1	179/726	1
Q2(31.5–42.0)	65/716	0.86(0.60–1.23)	170/716	0.84(0.68–1.05
Q3(42.0–55.2)	54/722	0.71(0.47–1.08)	132/722	0.65(0.50–0.84)
Q4(55.2–78.8)	48/718	0.70(0.44–1.13)	117/718	0.62(0.46–0.83)
Q5(>78.8)	34/719	0.60(0.33–1.07)	111/719	0.70(0.49–0.99)
*P* for trend		0.448		0.007
*P*_sex*HbGA_		0.005		0.008
**HbAA/HbGA**			
Q1(<0.89)	55/718	1	140/718	1
Q2(0.89–1.06)	45/717	0.95(0.64–1.41)	142/717	1.13(0.89–1.43)
Q3(1.06–1.24)	61/727	1.32(0.91–1.92)	131/727	1.05(0.83–1.34)
Q4(1.24–1.50)	53/721	1.27(0.87–1.87)	141/721	1.24(0.98–1.58)
Q5(>1.50)	54/718	1.61(1.09–2.39)	155/718	1.59(1.25–2.01)
*P* for trend		0.062		0.001
*P*_sex*HbAA/*HbGA*_		0.126		0.124

*Adjustments included age, sex, race, income, education, occupation, exercise, smoking, alcohol intake, energy, BMI, mean blood pressure, take medication for diabetes, and taking medication for hypertension and cholesterol. Case/N, number of case subjects/total; Q, quintile*.

The symbol * indicates the interaction between two variables.

Further, in line with the results of the total sample, results of subgroup analysis in the people with diabetes and pre-diabetes indicated that compared with the participants in the lowest quintile of HbAA/HbGA, the participants in the highest quintile of HbAA/HbGA had a greater risk of CVD mortality (*HR*: 1.92, 95% *CI*: 1.11–3.31 for diabetes and *HR*: 1.78, 95% *CI*:1.01–3.14 for pre-diabetes) and all-cause mortality (*HR*: 1.81, 95% *CI*:1.27–2.58 for diabetes and *HR*: 1.59, 95% *CI*: 1.14–2.20 for pre-diabetes), and sex was not a significant effect modifier of the above association (*p*_effectmodificationwithsex_ > 0.05) (Tables 3, 4). Meanwhile, no significant association between quintiles of HbAA or HbGA with mortality from CVD was observed among people with diabetes and pre-diabetes, respectively.

**Table 3:** Adjusted HRs for the associations between HbAA, HbGA, HbAA/HbGA and CVD or all-cause mortality in diabetes population.

	**CVD mortality**		**All-cause mortality**	
	**Case/N**	**HR (95%CI)**	**Case/N**	**HR (95%CI)**
**HbAA(pmoL/g Hb)**			
Q1(<34.9)	40/250	1	87/250	1
Q2(34.9–43.7)	24/249	0.68(0.40–1.15)	70/249	0.94(0.67–1.31)
Q3(43.7–56.1)	23/251	0.62(0.34–1.11)	60/251	0.81(0.56–1.18)
Q4(56.1–83.8)	21/248	0.67(0.33–1.34)	58/248	0.98(0.64–1.53)
Q5(>83.8)	24/249	1.23(0.55–2.77)	57/249	1.60(0.95–2.67)
*P* for trend		0.075		0.030
*P*_sex*HbAA_		0.057		0.114
**HbGA(pmoL/g Hb)**			
Q1(<30.7)	31/250	1	82/250	1
Q2(30.7–41.7)	32/249	0.95(0.57–1.59)	77/249	0.87(0.63–1.20)
Q3(41.7–54.2)	26/250	0.79(0.43–1.44)	66/250	0.70(0.48–1.02)
Q4(54.2–76.6)	20/249	0.74(0.36–1.53)	54/249	0.67(0.43–1.05)
Q5(>76.6)	23/249	0.81(0.37–1.80)	53/249	0.64(0.38–1.06)
*P* for trend		0.914		0.347
*P*_sex*HbGA_		0.042		0.023
**HbAA/HbGA**				
Q1(<0.85)	25/249	1	58/249	1
Q2(0.85–1.02)	26/250	1.23(0.70–2.14)	72/250	1.40(0.99–1.99)
Q3(1.02–1.19)	25/248	1.17(0.66–2.06)	62/248	1.23(0.85–1.77)
Q4(1.19–1.45)	24/251	1.15(0.65–2.03)	64/251	1.29(0.90–1.85)
Q5(>1.45)	32/249	1.92(1.11–3.31)	76/249	1.81(1.27–2.58)
*P* for trend		0.153		0.021
*P*_sex*HbAA/*HbGA*_		0.407		0.762

*Adjustments included age, sex, race, income, education, occupation, exercise, smoking, alcohol intake, energy, BMI, mean blood pressure, take medication for diabetes, and taking medication for hypertension and cholesterol. Case/N, number of case subjects/total; Q, quintile*.

The symbol * indicates the interaction between two variables.

**Table 4:** Adjusted HRs for the associations between HbAA, HbGA, HbAA/HbGA and CVD or all–cause mortality in pre-diabetes population.

	**CVD mortality**		**All-cause mortality**	
	**Case/*N***	**HR (95%CI)**	**Case/*N***	**HR (95%CI)**
**HbAA(pmoL/g Hb)**			
Q1(<37.1)	38/471	1	91/471	1
Q2(37.1–47.3)	31/472	0.79(0.48–1.32)	89/472	0.97(0.71–1.32)
Q3(47.3–60.2)	23/470	0.81(0.45–1.46)	64/470	0.89(0.62–1.28)
Q4(60.2–104.0)	30/473	1.53(0.82–2.84)	73/473	1.38(0.93–2.04)
Q5(>104.0)	14/468	1.66(0.67–4.14)	60/468	2.38(1.44–3.94)
*P* for trend		0.108		<0.001
*P*_sex*HbAA_		0.057		0.114
**HbGA(pmoL/g Hb)**			
Q1(<31.9)	33/474	1	92/474	1
Q2(31.9–42.2)	36/468	0.96(0.58–1.57)	98/468	0.93(0.69–1.25)
Q3(42.2–56.0)	33/472	0.91(0.52–1.60)	67/472	0.65(0.45–0.93)
Q4(56.0–79.8)	20/470	0.61(0.31–1.20)	60/470	0.61(0.41–0.91)
Q5(>79.8)	14/470	0.65(0.27–1.55)	60/470	0.85(0.55–1.39)
*P* for trend		0.579		0.023
*P*_sex*HbGA_		0.042		0.023
**HbAA/HbGA**			
Q1(<0.91)	27/469	1	75/469	1
Q2(0.91–1.09)	25/470	1.04(0.60–1.80)	76/470	1.05(0.76–1.45)
Q3(1.09–1.27)	32/474	1.52(0.90–2.57)	63/474	0.94(0.67–1.32)
Q4(1.27–1.55)	26/472	1.16(0.67–2.01)	81/472	1.22(0.89–1.69)
Q5(>1.55)	26/469	1.78(1.01–3.14)	82/469	1.59(1.14–2.20)
*P* for trend		0.186		0.014
*P*_sex*HbAA/*HbGA*_		0.407		0.762

*Adjustments included age, sex, race, income, education, occupation, exercise, smoking, alcohol intake, energy, BMI, mean blood pressure, and taking medication for diabetes and hypertension, take medication for cholesterol. Case/N, number of case subjects/total; Q, quintile*.

The symbol * indicates the interaction between two variables.

Additionally, the significant modification effects of sex on the relationship between HbAA and CVD mortality in the total sample, and the association of HbGA with mortalities of CVD and all-cause were observed among the total sample, and the samples of pre-diabetes and diabetes, respectively. We therefore examined this above association by sex, which is presented in the Supplementary Materials. In the total sample, the increased HbAA levels were negatively related with CVD mortality in men; whereas it was positively related with CVD mortality in women (Supplementary Table 1). Moreover, no significant relationship between HbGA and CVD mortality was observed in men (Supplementary Table 2); whereas an increase in HbGA was negatively associated with CVD or all-cause mortality in women (Supplementary Table 3). These findings indicated that compared with the ratio of HbAA and HbGA, the individual association of HbAA or HbGA with CVD mortality was more easily influenced by sex.

## Discussion

To our knowledge, the present study is the first to examine whether exposure to acrylamide is associated with long-term survival among people with hyperglycemia. In this study, we observed that higher levels of the ratio between HbAA and HbGA were related to greater CVD and all-cause mortalities among people with hyperglycemia, independent of other traditional risk factors of CVD. Moreover, this association was relatively robust, which was consistently observed among people with diabetes and pre-diabetes, respectively.

Currently, only a few cross-sectional studies and one prospective study have examined the association of the biomarkers of acrylamide with blood glucose, lipids, and CVD mortality among the general population (8, 17, 18). However, there are some contradictions among these results. The previous study found that a higher HbAA level was associated with lower fasting insulin and glucose (19), whereas the recent study found that increased urine biomarkers of acrylamide were associated with increased fasting blood glucose, probably through lipid peroxidation (20). Moreover, it has been found that HbGA is positively associated with serum triglycerides and low-density lipoprotein cholesterol, suggesting that a higher level of HbGA is associated with dyslipidemia (17), which contradicts the recent results regarding the association between whole blood HbGA and CVD, reporting that higher levels of HbGA were associated with the lower prevalence of CVD and CVD-mortality (21). The conflicting findings of these studies suggest that both HbAA and HbGA have limitations as markers of acrylamide exposure alone, whereas the present study integrates two acrylamide metabolites, HbAA and HbGA, by using a ratio of the two as biomarkers of acrylamide exposure for a more comprehensive evaluation. Furthermore, these conflicting results warrant a study focusing on people with diabetes or pre-diabetes to elucidate the association between exposure to acrylamide and long-term survival in this specific population.

In this study, although we observed the positive association of HbAA with CVD and all-cause mortalities in the total sample, this relationship could not be consistently observed among the people with diabetes or pre-diabetes, suggesting that this significant association in the total sample was likely driven by the relatively large sample size. In contrast, the association of higher levels of the ratio of HbAA and HbGA with mortalities of CVD and all-cause could be consistently observed among people with diabetes or pre-diabetes, suggesting that the combination of HbAA and HbGA was more important than focusing on the individual effect of HbAA or HbGA on the development of CVD among people with hyperglycemia. In fact, when acrylamide enters circulation, a proportion of acrylamide reacts with hemoglobin to form HbAA (22), and the other proportion of acrylamide can be metabolized by binding to glutathione (GSH) (23), which further can be oxidized into glycidamide by cytochrome P450 (24), and the epoxidation of hydrolyzate can largely convert glycidamide into non-toxic glycine amide due to the body's self-protective mechanism (25). The ratio of HbAA and HbGA probably reflects the balance of the detoxification metabolism in the body (4), and based on the findings of this study, the balance of metabolic networks for acrylamide may play a more critical role in the development of CVD among people with hyperglycemia.

Moreover, a series of the previous *Vivo*- and *Vitro*-experiments elucidated the mechanisms underlying the relationship between exposure to acrylamide and CVD mortality (26–28). In the past few years, there has been some progress in understanding the underlying impact of acrylamide on CVD, which is that acrylamide exposure may lead to oxidative stress and disturbance of physiological function (26, 28). A human study has found that the consumption of acrylamide-containing potato chips could cause significant changes in the levels of oxidative stress and inflammation response, probably increasing the risk of atherosclerosis (27). Moreover, zebrafish research has shown that acrylamide exposure could aggravate oxidation and degradation of low-density lipoprotein with increased production of reactive oxygen species (ROS) and cause dose-dependent fat accumulation in the liver (29). In addition, another animal study indicated exposure to acrylamide could cause a significant decrease in the GSH level (30) and stimulate oxidative stress, which might strike the blood vessels and exacerbate the progression of atherosclerosis (31). Further, *vitro* experiments also revealed that chronic exposure to acrylamide and glycidamide could induce accelerated endothelial aging, even at low concentrations (13). The results of our study showed that acrylamide exposure was significantly associated with CVD and all-cause mortalities, which could be partially supported by these studies above.

Further, we observed that among the people with diabetes, the participants whose ratio was ≥1.45 had greater CVD mortality compared with control; whereas among the people with pre-diabetes, the participants whose ratio was ≥1.55 had greater CVD mortality compared with control. These findings suggested that the ratio for increasing the risk of CVD mortality is lower in diabetes than in pre-diabetes, which adds to our understanding of how to develop an adequate range for the ratio among people with diabetes in the future. It has been reported that people with diabetes have higher levels of glutathione and cytochrome P450 2E1 than other people (32). Therefore, in people with diabetes, the circulating acrylamide tends to convert to HbGA, probably making the ratio of HbAA and HbGA significantly lower than in other people, and this mechanism supported the findings in this study.

This present study examined the relationship between exposure to acrylamide and the mortalities of CVD and all-cause, demonstrating the importance of the balance of metabolic networks for acrylamide among people with hyperglycemia. This association documented in our study had relatively strong robustness after adjusting for a range of important classical confounders. These findings also provided evidence for the impact of environmental exposure on the improvement of long-term survival for people with diabetes. Diabetes care professionals should be aware of the current findings from this study regarding the deleterious effect of exposure to acrylamide in daily life. This information is of importance in providing individualized treatment strategies in relation to avoiding environmental exposure for patients with diabetes. In addition, we recognized that this study had several limitations. First, we had adjusted a range of potential confounders, whereas the present study was still observational research, and some unmeasured confounding factors cannot be excluded. Second, this study was not able to distinguish between different types of diabetes. More research in the future is needed to examine this association in terms of type 1 and type 2 diabetes to provide more comprehensive evidence. Last, the measurement of exposure to acrylamide was only conducted at baseline. The studies with more frequent measurements may offer a more complicated blueprint of changes in the exposure to acrylamide and long-term survival among people with hyperglycemia.

## Conclusion

Exposure to acrylamide, which is indicated by the higher ratio of HbAA and HbGA in the whole blood, is related to higher CVD and all-cause mortalities among people with diabetes and pre-diabetes.

## Data Availability Statement

Publicly available datasets were analyzed in this study. This data can be found here: National Health and Nutrition Examination Survey https://www.cdc.gov/nchs/nhanes/index.htm.

## Author Contributions

CS and TH planned the work. JX, XS, HJ, and CH carried out the statistical analysis. HW wrote and reported the work. WW and WJ played critical roles in revising the manuscript. All authors made a significant contribution to this article, critically assessed and reviewed the paper, and approved the version to be published.

## Funding

This research was supported by the funding from the HMU Marshal Initiative Funding (HMUMIF-21011 to WJ, HMUMIF-21013 to WW) and the National Natural Science Foundation of China (81803227 to TH).

## Conflict of Interest

The authors declare that the research was conducted in the absence of any commercial or financial relationships that could be construed as a potential conflict of interest.

## Publisher's Note

All claims expressed in this article are solely those of the authors and do not necessarily represent those of their affiliated organizations, or those of the publisher, the editors and the reviewers. Any product that may be evaluated in this article, or claim that may be made by its manufacturer, is not guaranteed or endorsed by the publisher.
