# Supplementary material for: The Association Between Exposure to Acrylamide and Mortalities of Cardiovascular Disease and All-Cause Among People With Hyperglycemia
Source: Front Cardiovasc Med. 2022 Jul 18;9:930135. doi: 10.3389/fcvm.2022.930135 (PMC9339995; doi:10.3389/fcvm.2022.930135)
Supplement: Supplementary file 1 [file Table_1.DOCX]

**Supplementary table-1 Adjusted HRs for the associations between HbAA and** **CVD mortality in total hyperglycemia population in different sexes.**

| CVD mortality | | | | | |
| --- | --- | --- | --- | --- | --- |
| Men | | | Women | | |
|  | Case/N | HR (95%CI) |  | Case/N | HR (95%CI) |
| Q1(<37.0) | 41/392 | 1 | Q1(<35.4) | 38/333 | 1 |
| Q2(37.0-48.0) | 29/391 | 0.57(0.35-0.95) | Q2(35.4-45.1) | 28/330 | 0.82(0.51-1.33) |
| Q3(48.0-62.6) | 25/389 | 0.48(0.28-0.82) | Q3(45.1-56.0) | 24/330 | 0.77(0.46-1.27) |
| Q4(62.6-107.0) | 26/389 | 0.46(0.26-0.84) | Q4(56.0-86.6) | 17/330 | 0.91(0.54-1.52) |
| Q5(>107.0) | 28/388 | 0.78(0.40-1.56) | Q5(>86.6) | 12/329 | 1.76(1.01-3.07) |
| *P* for trend |  | 0.027 |  |  | 0.064 |

Adjustments included age, race, income, education, occupation, exercise, smoking, alcohol intake, energy, BMI, mean blood pressure, take medication for diabetes, take medication for hypertension, take medication for dyslipidemia.

Case/N, number of case subjects/total; Q, quintile.

**Supplementary table-2 Adjusted HRs for the associations between HbGA and CVD or all-cause mortality among people with hyperglycemia, diabetes and pre-diabetes in men**

| **HbGA(pmoL/g Hb)** | CVD mortality | | All-cause mortality | |
| --- | --- | --- | --- | --- |
|  | Case/N | HR (95%CI) | Case/N | HR (95%CI) |
| **Hyperglycemia population** | |  |  |  |
| Q1(<30.7) | 30/390 | 1 | 93/390 | 1 |
| Q2(30.7-40.7) | 31/394 | 0.95(0.57-1.58) | 90/394 | 0.90(0.67-1.21) |
| Q3(40.7-54.9) | 37/387 | 1.23(0.79-2.13) | 80/387 | 0.92(0.68-1.25) |
| Q4(54.9-79.2) | 28/389 | 1.14(0.67-1.93) | 70/389 | 0.98(0.71-1.35) |
| Q5(>79.2) | 23/389 | 1.15(0.64-2.06) | 71/389 | 1.24(0.89-1.72) |
| *P* for trend |  | 0.746 |  | 0.344 |
| **Diabetes population** | |  |  |  |
| Q1(<29.3) | 13/134 | 1 | 39/134 | 1 |
| Q2(29.3-39.3) | 15/126 | 0.95(0.45-2.03) | 47/126 | 1.07(0.69-1.66) |
| Q3(39.3-52.2) | 17/130 | 1.14(0.54-2.39) | 39/130 | 0.92(0.58-1.45) |
| Q4(52.2-76.4) | 12/129 | 0.93(0.41-2.11) | 34/129 | 0.99(0.61-1.59) |
| Q5(>76.4) | 15/129 | 1.02(0.45-2.31) | 33/129 | 0.96(0.58-1.59) |
| *P* for trend |  | 0.985 |  | 0.972 |
| **Pre-diabetes population** | |  |  |  |
| Q1(<31.5) | 16/264 | 1 | 49/264 | 1 |
| Q2(31.5-41.1) | 16/258 | 0.96(0.47-1.95) | 46/258 | 0.92(0.61-1.38) |
| Q3(41.1-56.1) | 21/262 | 1.64(0.83-3.21) | 41/262 | 1.02(0.67-1.56) |
| Q4(56.1-80.8) | 14/258 | 1.10(0.52-2.30) | 37/258 | 0.99(0.64-1.54) |
| Q5(>80.8) | 10/259 | 1.49(0.63-3.50) | 39/259 | 1.81(1.15-2.84) |
| *P* for trend |  | 0.457 |  | 0.033 |

Adjustments included age, race, income, education, occupation, exercise, smoking, alcohol intake, energy, BMI, mean blood pressure, take medication for diabetes, take medication for hypertension, take medication for dyslipidemia.

Case/N, number of case subjects/total; Q, quintile.

**Supplementary table-3 Adjusted HRs for the associations between HbGA and CVD or all-cause mortality among people with hyperglycemia, diabetes and pre-diabetes in women**

| **HbGA(pmoL/g Hb)** | CVD mortality | | All-cause mortality | |
| --- | --- | --- | --- | --- |
|  | Case/N | HR (95%CI) | Case/N | HR (95%CI) |
| **Hyperglycemia population** | |  |  |  |
| Q1(<32.6) | 39/333 | 1 | 88/333 | 1 |
| Q2(32.6-43.5) | 31/331 | 0.68(0.42-1.11) | 76/331 | 0.74(0.54-1.02) |
| Q3(43.5-55.7) | 21/328 | 0.49(0.28-0.85) | 55/328 | 0.60(0.43-0.86) |
| Q4(55.7-77.8) | 18/330 | 0.54(0.30-0.99) | 46/330 | 0.54(0.37-0.79) |
| Q5(>77.8) | 10/330 | 0.37(0.18-0.77) | 40/330 | 0.64(0.43-0.95) |
| *P* for trend |  | 0.027 |  | 0.010 |
| **Diabetes population** | |  |  |  |
| Q1(<322.5) | 22/121 | 1 | 45/121 | 1 |
| Q2(32.5-43.0) | 13/120 | 0.59(0.29-1.21) | 28/120 | 0.62(0.38-1.01) |
| Q3(43.0-55.2) | 8/119 | 0.37(0.15-0.88) | 25/119 | 0.58(0.34-0.97) |
| Q4(55.2-76.7) | 10/120 | 0.55(0.24-1.25) | 23/120 | 0.55(0.31-0.95) |
| Q5(>76.7) | 7/119 | 0.38(0.15-0.98) | 19/119 | 0.45(0.25-0.81) |
| *P* for trend |  | 0.134 |  | 0.054 |
| **Pre-diabetes population** | |  |  |  |
| Q1(<32.6) | 16/211 | 1 | 42/211 | 1 |
| Q2(32.6-43.7) | 19/211 | 0.83(0.41-1.68) | 48/211 | 0.78(0.50-1.20) |
| Q3(43.7-55.8) | 14/210 | 0.75(0.35-1.61) | 31/210 | 0.67(0.41-1.09) |
| Q4(55.8-78.3) | 6/211 | 0.37(0.13-1.01) | 22/211 | 0.46(0.27-0.80) |
| Q5(>78.3) | 4/210 | 0.47(0.15-1.46) | 22/210 | 0.62(0.51-1.50) |
| *P* for trend |  | 0.315 |  | 0.072 |

Adjustments included age, race, income, education, occupation, exercise, smoking, alcohol intake, energy, BMI, mean blood pressure, take medication for diabetes, take medication for hypertension, take medication for dyslipidemia.

Case/N, number of case subjects/total; Q, quintile.
