# Supplementary figures and images for: The Association Between Exposure to Acrylamide and Mortalities of Cardiovascular Disease and All-Cause Among People With Hyperglycemia
Source: Front Cardiovasc Med. 2022 Jul 18;9:930135. doi: 10.3389/fcvm.2022.930135 (PMC9339995; doi:10.3389/fcvm.2022.930135)

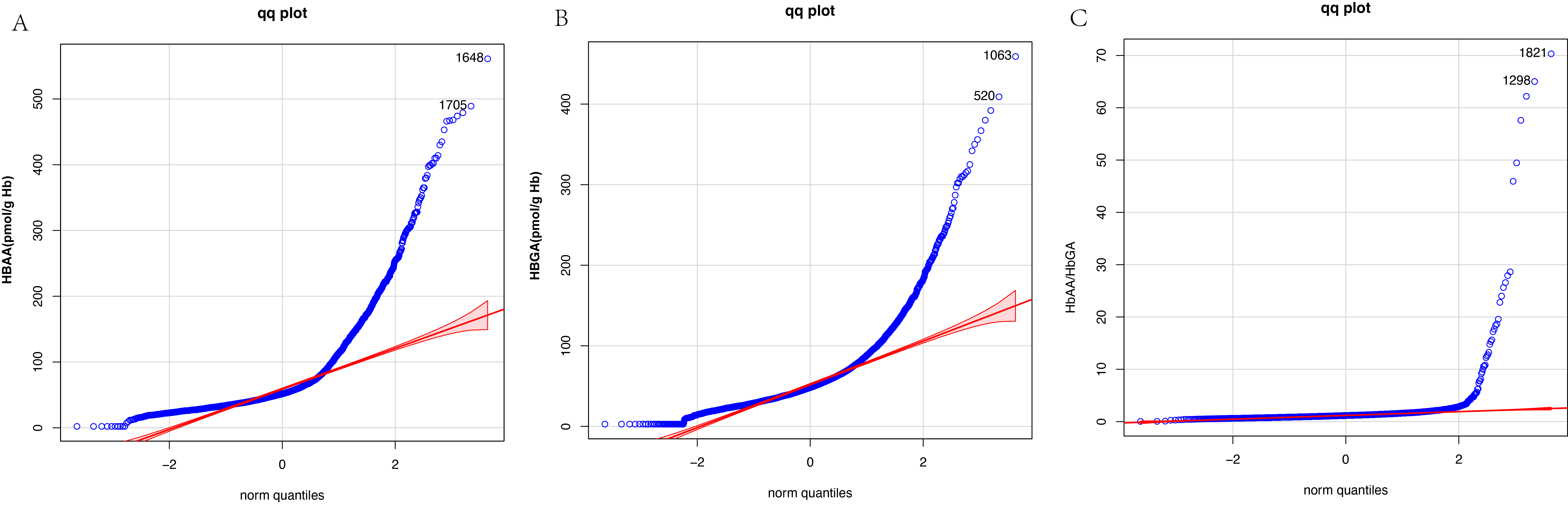

Supplement: Supplementary file 2 [file Image_1.JPEG]
